# Digital mental health and hidden support: a qualitative analysis of non-suicidal self-injury communities on TikTok

**DOI:** 10.3389/fdgth.2025.1645276

**Published:** 2025-10-03

**Authors:** Esther Martínez-Pastor, Marian Blanco-Ruiz, Sandra Feijóo

**Affiliations:** ^1^ Faculty of Communication Sciences, Rey Juan Carlos University, Fuenlabrada, Spain; ^2^ Department Health Sciences, University of Burgos, Burgos, Spain

**Keywords:** Non-Suicidal Self-Injury (NSSI), self-harm, digital mental health, online peer support, social media platforms, TikTok, adolescents and young adults, social media influence

## Abstract

This study examines the digital representation of Non-Suicidal Self-Injury (NSSI) on TikTok, with particular attention to the emergence of online communities and the communicative strategies users employ to share content while evading platform moderation. As TikTok becomes increasingly influential among adolescents and young adults, understanding how sensitive mental health topics like NSSI circulate on the platform is critical for developing effective digital health interventions. We conducted a qualitative content analysis of 400 posts referencing NSSI, collected using a mixed-method approach: 25.5% using TikTok's official API and 74.5% via the “For You” feed of a simulated account designed to mirror organic user experience. Posts were selected based on visual indicators (e.g., scars, tools), textual cues (e.g., hashtags, metaphors), and thematic references to emotional distress, recovery, or relapse. The analysis focused on user profile characteristics, linguistic strategies, and audiovisual aesthetics. Findings reveal a loosely structured yet emotionally resonant digital community characterized by subcultural codes, such as euphemisms, ambiguous hashtags, and stylized imagery. Despite content moderation policies, most accounts remained active and visible, with minimal enforcement of warnings or restrictions. While some posts portray NSSI as a coping strategy or seek to normalize the behavior, others subtly encourage recovery or offer indirect support. However, explicit messaging that discourages self-harm is notably rare. These dynamics suggest that TikTok unintentionally enables both the concealment and dissemination of self-harm-related content, functioning as a space for affective connection but also as a vector for potential normalization of harmful behaviors. The study underscores the need for targeted, ethically grounded prevention strategies that address not only the psychological functions of these communities but also the algorithmic infrastructures that sustain their visibility. These findings contribute to ongoing debates about digital mental health, platform responsibility, and the design of safer online environments.

## 1 Introduction

Non-Suicidal Self-Injury [NSSI], defined as the deliberate, self-inflicted damage of body tissue without suicidal intent and not for purposes that are socially accepted, is a significant health concern among adolescents and young adults (1). While NSSI is often hidden offline due to stigma, it has found visible expression in online spaces, especially on youth-centric platforms such as TikTok (2). Digital communities of young people who self-harm have been created on social media (3–5) where these young people feel safe enough to show and talk about self-harm, creating environments that promote this behavior (5–8). As one of the fastest-growing social media platforms globally (9), TikTok has evolved from its primary function as an entertainment site to serve as a powerful tool for identity expression, peer support, discussions, and portrayals of self-harming behaviors (10).

TikTok presents a distinctive appeal linked to its participatory and algorithm-driven environment, which encourages rapid content sharing, replication, and engagement. Over 80% of creators are under 25 (11), and its interactive features (e.g., duets, memes, sound overlays) foster “vernacular creativity” (12) and the formation of niche digital communities leading and participating in various activist initiatives aimed at global awareness, social change, civic politics, and mental health advocacy (2, 9, 13, 14). However, this design also allows for easy and casual engagement with problematic content, including misinformation (15), online toxicity (16), and mental health issues (17).

### 1.1 The double-edged sword of TikTok and mental health

There is a growing body of literature documenting the complex relationship between social media use and youth mental health. Some studies have reported negative outcomes linked to excessive screen time and harmful content exposure, such as depressive symptoms, anxiety, and problematic Internet use (18, 19). However, social media is also recognized as a valuable resource for enhancing mental health literacy, reducing stigma, and encouraging help-seeking behavior (20). Users can share their mental health struggles to build solidarity and community (21), while also encountering unregulated or misleading content from non-experts. Creators who are not trained psychotherapists post mental health-related content on social media, potentially substituting professional support for young people (16, 22). Furthermore, it has been found some types of social media content can reinforce problematic behaviors such as eating disorders (23) or Non-Suicidal Self-Injury [NSSI] (5, 24). Research on social media and self-harm [SH] among adolescents still debates whether social media use has a positive or negative impact on these behaviors (25). Weinstein (26) highlights this duality by likening the relationship between social technology use and well-being to a seesaw, where both positive and negative forces are constantly in play rather than fitting a simple “either/or” framework. Additionally, Shanahan et al. (27) describe how sharing self-harm experiences online can de-stigmatize mental health and model recovery trajectories, but only if done responsibly.

Recent findings underscore how the algorithm TikTok uses, specifically its “For You Page” (FYP), can intensify both positive and negative experiences. It offers unique opportunities not available in other online communities, fostering self-discovery and visibility, but also overexposes users to emotionally charged or triggering content (17). Although TikTok has acknowledged the negative uses of their platform and implemented content moderation, warnings, and resources specifically for users searching for suicide-related content (28), the practice of “algospeak” (i.e., coded language to evade censorship) enables communities to bypass restrictions by using hashtags and other codes to make their posts visible to knowing peers (29). For example, hashtags like “#blithe” or “#MySecretFamily” discreetly share SH content on Instagram (24). In the context of NSSI, users frequently use this encrypted communication or algospeak to share personal stories, images, or symbolic content that can normalize or romanticize the behavior (30–34). Adding to the debate, Lookingbill and Le (35) suggest that content moderation on social media can negatively affect young people, as they may feel marginalized and stigmatized if they cannot share content about self-harm [SH].

### 1.2 The relevance of online communities for young people

Studies show that adolescents turn to online communities to connect with people with similar interests (36), especially when stigma restricts open conversation. In online communities, young people find a space in which to share their discomfort without feeling judged and allows communication between young people about issues that they may be too ashamed to share in person (32). On the downside, it can also normalize maladaptive behaviors (8). For young people engaged in NSSI, TikTok offers a paradoxical space as it can provide the sense of belonging and validation they crave while simultaneously reinforcing harmful narratives through aestheticized or dramatized depictions of self-harm (30, 31). Exposure to this content can create an echo chamber effect, increasing the likelihood of engaging in self-harm behaviors (37, 38). There is also evidence that future self-harm may be motivated by the desire to publish content and maintain social reinforcement (39, 40).

Although some creators may use TikTok to promote resilience, share recovery journeys, or encourage professional help-seeking, others risk blurring the line between raising awareness and glamorizing NSSI. Most adolescents maintain positive expectations of social media, even when they have already experienced negative consequences (41). The desire for social belonging, which also underpins engagement in viral challenges (42), plays a central role in online community participation. Shared values and the use of domain-specific slang, such as algospeak, foster group identity and distinguish members from clueless outsiders (43). Moreover, online anonymity can increase conformity to perceived group norms by shifting the focus from personal to social identity (44). Therefore, being part of a TikTok community with the same interests and a clearly distinguished identity from others can help intragroup bonding and fulfill the need for social belonging for young people (42, 45), while at the same time posing a risk to reinforce the behavior.

### 1.3 The present study

This study focuses on TikTok to explore the representation of Non-Suicidal Self-Injury [NSSI] within this unique online social environment. TikTok was selected as a case study because of its massive youth user base (46), evolving moderation policies on mental health and self-harm (28), and potential role in shaping peer culture among adolescents. The overarching aim is to critically understand how NSSI is represented, shared, and potentially normalized in ways that escape platform moderation and shape youth identity, community, and online coping mechanisms. The following research questions guided the study:

• RQ1) What are the characteristics of TikTok profiles that post NSSI content?
• RQ2) What strategies do users employ to present NSSI content and build a community around it?
• RQ3) How do NSSI content creators circumvent moderation systems in TikTok?

It must be noted that TikTok's official policy prohibits content that promotes, glorifies, or normalizes Non-Suicidal Self-Injury [NSSI]. According to its Safety & Privacy Guidelines (28), the platform removes content that depicts or encourages self-harm, while allowing posts that aim to raise awareness or provide support, provided they do not include graphic imagery or methods. Despite these policies, our findings show that users often employ coded language and symbolic visuals, commonly referred to as “algospeak”, to evade moderation and maintain visibility within NSSI-related communities.

## 2 Method

A qualitative content analysis methodology was employed to explore how Non-Suicidal Self-Injury (NSSI) is communicated and represented on TikTok. The analysis followed a multi-step process inspired by thematic analysis and digital ethnography. First, an open coding phase was conducted to identify recurring patterns in visual, textual, and symbolic elements. These initial codes were then grouped into broader thematic categories through axial coding. Finally, interpretive analysis was applied to understand the socio-cultural meanings and community dynamics embedded in the content. While descriptive statistics (e.g., percentages) were used to illustrate the frequency of certain features, the primary aim was to uncover the communicative logic and affective structures underpinning NSSI-related content on the platform.

Given the nature of the platform, each TikTok post was treated as a unit of analysis, including the audiovisual content (e.g., music, voice-over, visual elements) and the accompanying textual content (e.g., captions, hashtags).

### 2.1 Data collection strategy

A mixed sampling strategy was employed to reflect both user-directed (search-based) and platform-directed (algorithmic) pathways for discovering content related to NSSI. Data collection took place between January and April 2024.

#### 2.1.1 Hashtag-based sampling via tikTok API

The first dataset was retrieved using the TikTok API, accessed via the TikApi library (47). Nine hashtags were selected based on prior literature identifying their prominence in online NSSI discourse: #Catscratchtwt, #Scars, #Cuttwt, #Beanstwt, #X_sh, #Sh, #Babycuts, #Shscars, and #Styrofoam (5, 48–50). These hashtags were chosen for their documented relevance to NSSI communities and behaviors. The initial search returned 875 posts, from which 102 (11.65%) were selected based on inclusion criteria described below.

#### 2.1.2 Algorithm-driven sampling via simulated account

To complement search-based results and account for TikTok's algorithmic recommendation dynamics, a simulated user account (@aiko3135) was created. This account was designed to replicate the behavioral patterns of an adolescent user with an emerging interest in NSSI-related content. The simulated account was designed to reflect the behavior of adolescents and young adults, who represent the majority of TikTok's user base. Although demographic data of content creators was not always explicit, profile indicators (e.g., language, imagery, self-descriptions) suggest that most users in the sample belong to this age group.

The simulation protocol involved passive scrolling, “liking” and saving NSSI-related videos over several sessions to train the recommendation algorithm. This approach aligns with prior research that leverages synthetic accounts to study how social media algorithms surface sensitive content (51–53).

The simulated account was active for a total of 90 days, during which it interacted with NSSI-related content to train the algorithm. This process resulted in the identification of approximately 1,200 posts, from which 298 were selected based on the inclusion criteria. Combined with the API-based sample, the final dataset consisted of 400 posts.

#### 2.1.3 Inclusion and exclusion criteria

Posts were included if they contained explicit or implicit references to NSSI, operationalized as visual indicators (e.g., scars, fresh wounds, tools associated with self-injury), textual cues in captions or hashtags, thematic references (e.g., emotional pain, self-harm, recovery, relapse). Duplicates, off-topic content, or videos lacking sufficient contextual information were excluded (see Table 1).

**Table 1:** Inclusion and exclusion criteria.

Inclusion criteria	Exclusion criteria
Posts published on TikTok	Content from other platforms (e.g., Instagram, X.com, YouTube)
Videos in English or Spanish	Videos in other languages not understood by research team
Posts with explicit or implicit references to non-suicidal self-injury (NSSI), including: Visual indicators, textual references in captions, hashtags, or overlays, and thematic references to emotional distress or coping through self-injury.	Posts unrelated to NSSI or containing exclusively suicidal content without self-harm references.
Original, edited, or repurposed videos created by users, containing visual, textual, or symbolic representations related to NSSI.	Content unrelated to the topic (e.g., lifestyle, humor, beauty tutorials).
Public individual user accounts.	Institutional, commercial, or private accounts.
Posts accessible at the time of collection and sufficiently contextualized for analysis.	Deleted, suspended, private, or context-deficient posts (e.g., no caption, visuals, or relevant hashtags).

Following this criteria, the final sample consisted of 400 posts, of which 25.5% were collected via the API and 74.5% via the simulated account's “For You” feed.

### 2.2 Content analysis procedure

A structured codebook of 103 variables was developed to guide the qualitative content analysis. These variables were derived from a comprehensive review of prior research on NSSI communication, mental health in social media, and media representation of self-harm (54, 55), as well as the authors’ prior work (4). Variables captured multiple dimensions of each post, including:

• Visual content: presence of scars, blood, or tools.
• Textual markers: use of recovery vs. pro-NSSI language.
• Narrative tone: expressions of despair, romanticization, normalization, or encouragement of self-injury.
• Affective cues: expressions of emotional pain, isolation, or solidarity.

Coding was performed by two trained researchers following a two-phase intercoder training protocol to ensure consistency and minimize subjectivity (56). Intercoder reliability was assessed during a pilot coding session using a subset of 30 posts, achieving an agreement rate above 85%. Discrepancies were resolved through discussion and refinement of the coding schema. Final coding was carried out independently and results were cross-validated by both coders.

The combined use of data mining and algorithmic exposure allowed for the identification of digital communities and content circulation patterns that reflect how TikTok users engage with NSSI-related material. As Airoldi (57) emphasizes, digital ethnography not only examines user practices and platform mechanics but also investigates the emergent social and cultural phenomena that manifest within networked spaces. In this sense, our content analysis does not merely catalogue representations of NSSI, but seeks to uncover the communicative logic and community dynamics that sustain such representations on TikTok.

Each post was saved as a video file and accompanied by a metadata sheet including the caption, hashtags, and user profile information available. Screenshots were captured when relevant to facilitate visual analysis. Transcripts were created for videos with spoken or written text, and all materials were stored in a secure, encrypted institutional server. This transformation of data into analyzable formats allowed for coding and thematic interpretation.

### 2.3 Ethical considerations

All information and metadata analyzed were obtained from public TikTok profiles and did not include any personally identifiable information. The data were securely stored on the servers of the leading author institution and encrypted to ensure that only the researchers involved in the data analysis had access. This approach aligns with the ethical guidelines for conducting research on publicly accessible online content while safeguarding user privacy (58). This project was approved by the Ethics Committee of Rey Juan Carlos University (no: 0802202307023). Additionally, no examples of TikTok screenshots where the person can be recognized are included to preserve the privacy of users, even though all content has been retrieved from public profiles.

## 3 Results

This section presents the findings from the qualitative content analysis, organized into three main areas: profile characteristics (RQ1), textual analysis, and audiovisual analysis of the identified content. Across these dimensions, we highlight how TikTok users create and share NSSI content (RQ2) while concealing it from platform moderation (RQ3).

### 3.1 Profile characteristics of accounts sharing NSSI content

Accounts were identified as related to NSSI through a process combining keyword detection (e.g., references to “self-harm” or associated metaphors in bios or captions), qualitative indicators (e.g., use of metaphors such as “beans”, “barcodes”, or red color codes), and the presence of visual content displaying either fresh or healed wounds. While the academic literature uses NSSI as a descriptor for this type of content, users themselves seem to employ the term “self-harm” [SH]. Therefore, we adopted the term SH when describing user-generated content as a synonym for NSSI.

Based on the classification, 53% of the analyzed posts originated from profiles explicitly centered on SH-related content, whereas 44.75% came from profiles that were not framed as such but regularly posted SH material.

Although TikTok's community guidelines restrict SH-related content, 97.5% of the analyzed accounts were still active at the time of data collection, suggesting that much of this content successfully circumvented moderation protocols and control filters.

Regarding the identity of users who posted the analyzed content, 53.75% employed anonymous or fictional profile images, while 43.5% used personal photos. Based on images and available profile data, 44.5% of users presented as female, 11.5% as male, and 41.5% were unidentifiable in terms of gender expression or omitted gendered references entirely. The language distribution across profiles was as follows: 52% in English, 42.5% in Spanish, and 2.75% in other languages.

### 3.2 Communicative strategies in self-harm content

#### 3.2.1 Textual choices

Users strategically employed indirect, coded, or euphemistic language. None of the analyzed posts carried TikTok-issued sensitive content notifications. This may be related to the indirect or coded language that users employ. For instance, 82.2% of the post titles made no mention of mental illness or disorder, and explicit autobiographical references were relatively rare, with only 25.8% referencing their own experience in the title and 28.4% doing so in the post text. Instead, users often adopt generic or impersonal language, such as second or third-person phrasing. References to SH were frequently metaphorical or euphemistic (e.g., “tiger stripes” to refer to scars), which may serve to avoid detection and signal group membership. Only 4.2% referred to wounds as real in the title, while 95.8% did not mention wounds at all.

#### 3.2.2 Emojis and emotional cues

Emojis played a symbolic role in framing the content, adding an emotional and aesthetic layer. Emojis were included in 19.3% of the content analyzed in the title. Symbols that could be interpreted as “love” (hearts, kisses) were the most common (8%), followed by emojis associated with happiness (4%), animals (3%), and sadness (less than 1%). There even seems to be a positive reinterpretation not only by using these emojis but also by the accompanying narrative and hashtags. For example, one post featured stars drawn around scars with hashtags such as #drewstarsaroundmyscars and #malikandlikay, framing the content as affectionate or gentle rather than alarming.

#### 3.2.3 Hashtags and engagement

Hashtag strategies play a critical role in how NSSI-related content circulates on TikTok. Specific coded expressions or algospeak, such as “tiger stripes”, appear to serve dual functions: they enhance the discoverability of content for in-group users while circumventing moderation mechanisms that could lead to content removal. Additionally, 55.5% of the analyzed posts included direct prompts for engagement (e.g., “follow me”), suggesting a strong emphasis on community-building and social connectivity. This type of content often goes beyond algorithmic visibility or network logic, reflecting deeper social dynamics. For example, some posts combine insider terminology with positive emotional cues (such as depictions of users expressing joy or relief upon recognizing others with visible signs of self-injury) thereby reinforcing mutual identification and emotional solidarity within the self-harm community.

### 3.3 Evasion of content moderation

The vast majority of content successfully bypassed TikTok's moderation protocols. Notably, none of the analyzed posts displayed platform-generated sensitivity warnings, despite the presence of SH-related themes.

#### 3.3.1 Audiovisual tactics

Most of the analyzed videos (85.3%) were original content recorded by the users themselves, predominantly featuring real people. The remaining 14.8% used clips from fictional sources, such as TV shows or films, and 20% repurposed video fragments from other users.

Aesthetic or fictionalized alternatives were sometimes used to reference SH practices to avoid sharing real content, including clips from video games or anime (13.3% of videos), or stylized content with a cute (4.5%) aesthetic characterized by themes involving teddy bears, kittens, hearts, and pastel colors. Other minoritarian tactics employed to communicate on TikTok while evading controls are “Asian-inspired” themes (2.3%) and the use of memes (1.2%).

Text overlays were a dominant feature (90.2%). Of these, 33.1% used red-colored text, a practice often identified by users themselves with hashtags like #redtexttoidentify. These texts were written from a first-person perspective in 63.4% of the videos, and 74% explicitly described alleged personal experiences of the poster.

#### 3.3.2 Visual and symbolic strategies

Despite the sensitive nature of the content, very few videos contained graphic imagery. Only 0.8% showed blood, but scars were more common, as 18% of videos showed healed wounds. Regarding written mentions of wounds in the videos, 59.2% referred to self-harm in some manner. Tools such as blades, lighters, or pencil sharpeners appeared in 13.5% of the videos, and in 3.1% of the videos, they were romanticized using filters, music, or symbolic captions. In 12.5% of the videos, there was a positive attitude toward wounds. Conversely, a smaller number of posts (8%) featured recovery-oriented visuals, such as screenshots from apps and graphs tracking days without SH. Captures are usually taken from apps originally used for addictive behaviors.

In summary, visual and symbolic strategies show a balance between self-expression, peer engagement, and strategic concealment, often using platform-native tools (e.g., aesthetics, hashtags, text overlays) to create a subcultural language that signals belonging while evading censorship.

### 3.4 Emergent themes: self-harm post as emotional regulation and peer support

An emergent theme identified during data analysis was the framing of self-harm (SH) as a coping mechanism or tool for emotional regulation. Several posts explicitly described SH as helpful during moments of emotional distress, with some users defending past episodes of self-injury as functional or necessary in the context in which they occurred. These narratives were often met with sympathetic responses from other users, who contributed their own experiences in the comments, expressing empathy or identification with the emotional state described in the original post.

Quantitatively, 10.9% of the posts contained explicit statements that SH helped the user feel better, while an additional 10.9% referenced either mood disturbances or the physical pain associated with self-harming. A smaller but significant portion of users (3.9%) used the platform to seek advice or opinions from others regarding SH, sometimes posing direct questions to content creators or the broader community. This pattern suggests that beyond its expressive function, NSSI content on TikTok may serve as a peer-to-peer support space where users seek validation, normalization, or shared coping strategies.

Regarding mentions of ending self-harming behavior, 12.5% of the analyzed posts discussed desistance from the practice, typically framed as an aspiration or ongoing process. However, only 2.5% of the posts actively encouraged avoiding SH. Most content did not clearly endorse or condemn the behavior, but the messages tended to convey emotional pain instead. In fact, 89.3% of the titles contained no positive framing of SH, and 92% did not express any negative evaluations either.

Requests for help were rare, as only 1.3% of posts explicitly mentioned seeking support. However, social references were frequent (for example, 25.3% mentioned friends or family in some way). Some users directly addressed TikTok's moderation in captions like “block don't report”, “no flop”, or “TikTok no me banees” (“TikTok don't ban me”), suggesting awareness of the policies and trying to discourage others from reporting their sensitive content, requesting that those who are uncomfortable with their content block them instead of reporting them. Other posts illustrated the tension between visibility and stigma. For example, a video entitled ‘POV: you've got SH scars in summer' showed the anticipated judgement of others for wearing fashionable jeans with ripped holes showing the scars of self-harm, but ended with a message of empowerment: ‘wear what you want to wear’.

Table 2 below provides a summary of the main communicative strategies identified across the dataset, including textual, visual, and engagement tactics. This synthesis supports the thematic patterns discussed in the results and serves as a bridge to the interpretive discussion that follows.

**Table 2:** Summary of communicative strategies in NSSI-related TikTok posts.

Category	Strategy	Example/Frequency
Textual strategies	Euphemisms	e.g., “tiger stripes” for scars
	Hashtags	e.g., #beanstwt, #shscars, #redtexttoidentify
	Indirect language	82.2% did not mention mental illness explicitly
	Autobiographical references	25.8% in title, 28.4% in post text
	First-person perspective	63.4% of videos
Visual strategies	Scars	18% of videos showed healed wounds
	Blood	0.8% of videos
	Tools	13.5% showed blades, lighters, etc.
	Red text overlays	33.1% of videos
	Cute aesthetic	4.5% of videos
	Anime/Game clips	13.3% of videos
Engagement strategies	Calls to follow	55.5% of posts
	Comments for empathy	Users shared experiences and support
	Avoiding moderation	Captions like “TikTok don't ban me”
	Recovery visuals	8% of posts showed sobriety apps

This table categorizes the main communicative strategies identified in the qualitative analysis of 400 TikTok posts related to Non-Suicidal Self-Injury (NSSI). It includes textual, visual, and engagement strategies used by users to express emotional distress, foster community, and evade platform moderation. Frequencies are based on the coded sample and illustrate the prevalence of specific tactics such as euphemisms, red text overlays, and calls for interaction.

## 4 Discussion

This study examined TikTok posts related to Non-Suicidal Self-Injury (NSSI), focusing on whether a digital community forms self-harm practices and the communicative strategies used by content creators not only to engage with others and also avoid platform moderation. The results suggest that despite platform guidelines (28), NSSI-related content remains accessible, and creators frequently use indirect, symbolic, or stylized references to evade moderation.

A considerable portion of posts (73%) featured real individuals, though explicit images of self-harm were rare. Instead, creators utilized symbolic or euphemistic codes, such as terms like “tiger stripes” or visual motifs with a “cute aesthetic” to signal distress in ways recognizable to those familiar with the subculture. These findings align with prior research documenting platform-specific forms of communication that allow users to maintain visibility while circumventing content removal (29).

Regarding the characteristics of TikTok profiles posting NSSI content (RQ1), our findings indicate that some users actively construct a digital identity centered on self-harm. These users often reference NSSI in their bios and describe their accounts as spaces for emotional expression or sharing personal struggles. This behavior aligns with prior research showing that NSSI can serve as a coping mechanism for emotional regulation (59) and reflects principles of social identity theory (45). By seeking emotional connection with others experiencing similar distress, users transform individual suffering into a shared identity (42). This group affiliation fosters a sense of belonging and may become a core part of their online persona. The online environment further enables expressions of identity that may be suppressed in offline contexts due to a disinhibition effect (60).

Based on the results, 53% of the analyzed posts originated from profiles explicitly centered on SH-related content, often referencing emotional relief. This pattern reflects psychological models that conceptualize NSSI as a maladaptive yet functional strategy for short-term emotional regulation (61). However, these models also highlight the long-term risks and potential dependency associated with repeated self-harm (62, 63).

Regarding RQ2 (What strategies do users employ to present NSSI content and build community?), our data reveal a consistent use of coded language and shared symbols to foster group recognition and cohesion. Hashtags like #tigerstripes, #beanstwt, or #shscars act as in-group markers that signal community affiliation. These practices help establish shared norms and facilitate affective bonding, consistent with theories of online subcultures (43, 64). Many users shared personal experiences or expressed empathy towards others in the comments, suggesting that NSSI-related content often serves as a way to connect with peers. A loosely structured, emotionally driven community appears to form around shared experiences of distress and coping. As Tajfel et al. (45) noted, such group identification can strengthen social ties, especially for individuals who feel marginalized and stigmatized by the broader society. However, the degree to which these communities provide support vs. normalizing NSSI remains complex (25, 26).

Interestingly, RQ2 and RQ3 (How do NSSI content creators circumvent moderation systems in TikTok?) have at least one shared answer. Users appear highly aware of moderation practices and tailor their content accordingly, avoiding explicit language or images. The use of a distinct communicative style, often referred to as algospeak (29), allows creators to evade moderation while also reinforcing community identity. While the term “self-harm” occasionally appeared, content generally avoided explicit reference to suicide and was more subtle than the explicit representation of SH found on Twitter (5). This points to the development of a coded language specific to the NSSI TikTok space, deepening users’ sense of social identity and community belonging. The frequent use of red text, for example, appears to hold symbolic meaning within the SH community. These stylistic choices reflect the emergence of a subculture with its own norms and domain-specific slang, designed to build in-group cohesion and alienating outsiders (43, 64).

The symbolic and indirect nature presentation of content suggests a deliberate strategy to bypass content moderation and aligns with prior observations of users creatively reframing mental health content on TikTok (16, 22). Contrary to what might be expected in a community with alleged mental health issues (61–63), emojis depicting sadness or shame were rarely used. This may reflect a strategy to obscure emotional vulnerability or aesthetic preferences within this subculture. Instead, emotional themes were conveyed through a positive narrative, including visuals, overlays, and carefully curated hashtags. However, the use of apps designed to track sobriety among individuals recovering from addiction suggests that some creators possess metacognitive awareness of self-harm as both a harmful behavior, but also a practice that is difficult to overcome. This echoes findings on female smokers who use tobacco as a coping mechanism, and first seek support for developing healthier emotional regulation strategies when attempting to quit (65). Correspondingly, interventions attempting to tackle NSSI may be more effective if they focus on promoting healthier coping strategies rather than solely aiming to eliminate self-harming behaviors.

On the other hand, our data suggest that the algorithm reinforces exposure to NSSI-related content once a user begins interacting with it. After the research account engaged with SH-related posts by liking or saving them, its feed began to show more similar content. This pattern supports previous research indicating that recommendation systems can unintentionally promote harmful content through engagement loops (17). Additionally, some users encouraged others to like or follow their posts to increase visibility, showing a strategic understanding of how to navigate and amplify content within the platform.

These findings contribute to the growing literature highlighting the double-edged nature of online peer-to-peer mental health content. On the one hand, NSSI communities may provide a safe space for emotional expression and peer connection. On the other, they may also normalize maladaptive coping strategies or create echo chambers that discourage proffesional help-seeking (8, 38). Only 2.5% of posts in our sample explicitly discouraged self-harm. However, some users did try to offer peer support or attempted to reframe their scars as part of a personal recovery narrative.

Despite TikTok's official Well-Being Guides and partnerships with mental health organizations, our findings echo earlier concerns about the limited impact of institutional accounts (5, 66). In contrast, user-generated content receives substantially more attention, highlighting a disconnect between official messaging and user engagement. This suggests that public health campaigns may need to better align with the aesthetics, tone, and interactive styles appreciated by users. As many young people now turn to social media as an initial source of mental health information (67), these communication gaps are particularly concerning. Furthermore, researchers advocate for more proactive and transparent actions by administrations and platforms, such as TikTok, which would involve greater transparency regarding content suggestion algorithms. For example, Milton et al. (17) proposed design changes to enhance mental health support and suggested further investigation into social computing within algorithm-driven environments. There is also a lack of data on the effects of traumatic or “triggering” content, the actual use and effectiveness of TikTok's mental health resources, or the potential benefits of positive, supportive messages on young users’ mental health (30).

Regarding the limitations of this study, the use of a simulated account introduces certain biases, as algorithmic exposure may not fully reflect the experience of real users. The geolocation of the account in Spain likely influenced content language and cultural framing, limiting the generalizability of findings to broader TikTok communities. Furthermore, the performative nature of online identity complicates the interpretation of intent behind posts, and without user interviews, conclusions about motivations remain tentative. This study is limited by its reliance on a simulated account, which may not fully replicate the experience of real users. The lack of engagement with actual users further restricts our ability to assess the nuances behind content creation and sharing or infer the intent of creators. While a simulated account provides valuable observational insights, it cannot capture the subjective experiences or emotional states of those posting NSSI-related material.

Despite these limitations, the study offers important insights for digital mental health interventions and platform governance. The findings highlight the need for moderation systems that can recognize and respond to subcultural codes, as well as for public health messaging that aligns with the aesthetic and communicative preferences of youth communities online. Future research should move beyond content-analysis to center lived experiences and investigate how these online interactions influence offline well-being, particularly among young people at heightened risk of self-injury. Additionally, the influence of these communities on help-seeking behavior and long-term mental health outcomes should be examined, as well as platform-level interventions that balance freedom of expression with user safety.

## 5 Conclusion

This study investigated how self-harm is represented on TikTok, revealing a loosely formed yet affectively resonant digital community in which users construct identities rooted in distress and coping. Leveraging specific hashtags, subcultural aesthetics, and semi-coded language like metaphors or red-tinted text, creators can generate group belonging while evading moderation. References to recovery tools (i. e., apps to track sober behavior) further indicate a user base simultaneously aware of risks and invested in navigating them. Algorithmic exposure patterns suggest that engaging with NSSI content leads to increased visibility, further revealing the dual role of TikTok as both a site of peer support and a space where maladaptive behaviors can be subtly reinforced. Recommendation systems and moderation policies must adapt to the coded vernacular of NSSI communities.

Overall, while this digital community may provide validation, emotional release, and a sense of belonging for some users, it also normalizes self-injurious behaviors and raises complex questions for prevention efforts. Effective interventions must be grounded in an understanding of platform-specific cultures and the affective needs these communities fulfill, particularly among young people. Addressing this challenge requires interdisciplinary collaboration across youth psychology, communication and media studies, platform governance, and online-community research to develop empathetic, evidence-based digital-mental-health strategies. Future work should explore how digital platforms can respond more effectively and how public health strategies can become responsive to platform-specific cultures and communicative practices.

## Data Availability

The raw data supporting the conclusions of this article will be made available by the authors, without undue reservation.
